# Non-verbal IQ and change in restricted and repetitive behavior throughout childhood in autism: a longitudinal study using the Autism Diagnostic Interview-Revised

**DOI:** 10.1186/s13229-021-00461-7

**Published:** 2021-08-14

**Authors:** V. Courchesne, R. Bedford, A. Pickles, E. Duku, C. Kerns, P. Mirenda, T. Bennett, S. Georgiades, I. M. Smith, W. J. Ungar, T. Vaillancourt, A. Zaidman-Zait, L. Zwaigenbaum, P. Szatmari, M. Elsabbagh, Pathways Team

**Affiliations:** 1 Department of Neurology and Neurosurgery, McGill University, Montreal, Canada; 2 King’s College London, London, UK; 3 University of Bath, Bath, UK; 4 McMaster University, Hamilton, Canada; 5 University of British Columbia, Vancouver, Canada; 6 Dalhousie University, IWK Health Centre, Halifax, Canada; 7 Program of Child Health Evaluative Sciences, The Hospital for Sick Children Research Institute, and the Institute of Health Policy, Management and Evaluation, University of Toronto, Toronto, Canada; 8 University of Ottawa, Ottawa, Canada; 9 Tel Aviv University, Tel Aviv-Yafo, Israel; 10 University of Alberta, Edmonton, Canada; 11 Centre for Addiction and Mental Health, Hospital for Sick Children, University of Toronto, Toronto, Canada

**Keywords:** Autism, Longitudinal, Restricted, Repetitive, Behaviors, Interest, Intelligence, ADI-R, Wechsler

## Abstract

**Background:**

Restricted and repetitive behavior (RRB) is one of the characteristic features of Autism Spectrum Disorder. This domain of symptoms includes a broad range of behaviors. There is a need to study each behavior individually to better understand the role of each in the development of autistic children. Moreover, there are currently no longitudinal studies investigating change in these behaviors over development.

**Methods:**

The goal of the present study was to explore the association between age and non-verbal IQ (NVIQ) on 15 RRB symptoms included in the Autism Diagnostic Interview-Revised (ADI-R) over time. A total of 205 children with ASD were assessed using the ADI-R at time of diagnosis, at age 6 years, and at age 11 years, and with the Wechsler Intelligence Scales for Children—Fourth Edition (WISC-IV) at age 8 years.

**Results:**

The proportion of children showing each RRB tended to diminish with increasing age, except for *sensitivity to noise* and *circumscribed interests,* where the proportion increased over time. Although there was no significant main effect of NVIQ, there was a significant interaction between age and NVIQ. This was mainly driven by *Difficulties with change in routine*, for which higher NVIQ was associated with the behavior remaining relatively stable with age, while lower NVIQ was associated with the behavior becoming more prevalent with age.

**Limitations:**

The study focused on the presence/absence of each RRB but did not account for potential changes in frequency or severity of the behaviors over development. Furthermore, some limitations are inherent to the measures used. The ADI-R relies on parent report and hence has some level of subjectivity, while the Wechsler intelligence scales can underestimate the intellectual abilities of some autistic children.

**Conclusions:**

These results confirm that specific RRB are differentially linked to age and NVIQ. Studying RRB individually is a promising approach to better understanding how RRB change over the development of autistic children and are linked to other developmental domains.

**Supplementary Information:**

The online version contains supplementary material available at 10.1186/s13229-021-00461-7.

## Background

Restricted and repetitive behaviors (RRB) are central to the phenotype and diagnosis of Autism Spectrum Disorder (ASD) (DSM-5 1). RRB are considered “positive” symptoms of autism as they include behaviors that the person does more often or differently, instead of an absence of behaviors, like many described in the social-communication domain [2]. These symptoms include stereotyped and repetitive movements (e.g., finger mannerisms, arm movements or hand flapping), stereotyped speech (e.g., echolalia), insistence on sameness (e.g., rigid adherence to certain routines or rituals), intense interests in specific areas such as horses, animals or public transport maps, and special skills (e.g., calendar calculation or memory for routes) [1, 3, 4]. RRB are observed in autistic children of all intelligence levels but they appear to vary in degree depending on age and cognitive level [2, 5].

Despite their higher prevalence in autism, RRB are also observed in populations diagnosed with other conditions such as intellectual disability, Tourette syndrome, obsessive–compulsive disorder [6–8] and in typically developing children [i.e. 6, 7, 9]. These behaviors are perceived differently in developmental conditions and in typical development [10]. In autism it was proposed that some types of behavior that are often labelled as “lower order” RRB would be linked to the lower cognitive level of the child and not specific to autism [11], while others, generally labelled as “higher order’’ RRB may be an inherent part of autistic development [2, 12, 13]. The latter reflects manifestations of the way autistics perceive and process information [14, 15]. Attempts to understand the role of these behaviors and their relation to age in typically developing children could be informative for clarifying the role of RRB in autism [10]. For example, early in infancy, stereotyped motor behaviors are thought to be intrinsic to the development of complex functional motor behaviors in typically developing children [10, 16, 17]. These stereotypies are also thought to serve a communication function since they are more frequently observed during child-caregiver interactions and diminish as verbal abilities develop in typically developing children [17–19]. Furthermore, for typically developing children, “higher order” repetitive behaviors such as rituals, are considered a way to adapt to one’s environment such that by repeating, classifying, and ordering, the brain is able to organize information from the environment [20]. Similar patterns of development could characterize autistic children with normal intelligence, but more research is needed to disentangle the effects of age and cognitive level on RRB.

In order to understand the role of RRB in autism, research to date has taken three approaches. One approach is to study the domain of RRB symptoms as a whole, combining all individual behaviors and investigating whether presenting more or fewer symptoms overall is positively or negatively related to other domains of functioning, such as intelligence, and how the whole RRB domain changes with age. The second approach is to group individual RRB into categories, usually derived from factor analysis, and the third is to study each RRB individually.

When studied as a whole, RRB increase throughout preschool years [5] but diminish later in childhood [21, 22]. This is especially the case for autistic individuals with higher cognitive and language abilities [23]. Moreover, the presence of more RRB is generally linked to lower adaptive functioning [24], language abilities [25], and intelligence [26].

A different pattern of results emerges when RRB are investigated using factor analyses. The number of factors usually identified depends on the specific RRB measure and the age range of the sample. For example, using the Autism Diagnostic Interview-Revised (ADI-R, 27), when participants older than 3 years are included, a third factor, *circumscribed interests (CI),* emerges in addition to the *Repetitive and Sensory Motor (RSM)* and *Insistence on Sameness (IS)* factors (e.g., 28). Using the Repetitive Behavior Scale—Revised (RBS-R, 29), some researchers have identified a three-factor model [30], whereas others have found five- [28, 30] or six-factor models [31].

The links between the categories of RRB derived from factor analysis and other developmental domains are somewhat inconsistent (see: 23 for a review). For example, the score on the RSM factor derived from the ADI-R was shown to be negatively correlated with adaptive functioning [3], while none of the factors derived from the RBS-R were shown to correlate to developmental level [30]. The *IS* factor is often not correlated with IQ [12, 28], but the corresponding factor derived from the RBS-R is negatively correlated with NVIQ [24]. Taken together, studying RRB as a whole or even as factors has not revealed a consistent pattern of results across studies. A more fine-grained exploration of the RRB domain is warranted to clarify the nature of the links between developmental domains and the different behaviors included in the RRB domain. To date, few researchers have looked at individual RRB. Bishop, Richler [2] explored the association between each of the 15 RRB items from the ADI-R, age, and NVIQ in a large cross-sectional study of 830 autistic children ranging in age from 3 to 11 years. Although the association between NVIQ and the prevalence of specific RRB was generally negative (i.e., the behavior was more prevalent in groups of children with lower NVIQ), two specific RRB were more prevalent in children with higher NVIQ, namely *Compulsions/rituals* and *Circumscribed interests*. These positive and negative associations with NVIQ tended to become stronger with age. As for the severity of each behavior, it was either not associated with NVIQ or was related in the same fashion as was prevalence [2].

This approach illustrates that taking a more global or factor analytic approach to RRB could lead to specific behaviors being combined despite their differential associations with intelligence, adaptive behavior, language level, or age [2, 26]. These findings also suggest that the presence of some individual behaviors may be more strongly linked to developmental delay or cognitive impairment, while others might be more specific to the autism phenotype and development [2, 13, 32]. The presence of a specific RRB early in development might not have the same implications as the presence of this same behavior later. Indeed, the presence of a behavior at a young age may not necessarily be linked to NVIQ at that time in development. However, if that same behavior is still present as the child gets older, it can indicate a lower IQ [2]. An approach using individual behaviors is thus warranted.

To date, cross sectional studies have provided only snapshots of development across rather than within individuals over time. In the current study we build on previous work by Bishop and Richler [2] by longitudinally examining specific RRB included in the ADI-R in relation to age and NVIQ in an inception cohort of children followed from age of diagnosis to around 11 years. This longitudinal approach, allowed us to investigate change in individual RRB over time and their association with NVIQ in the same children, across three timepoints covering both preschool and school-age in a large autism cohort.

## Methods

### Participants

Participants for this study were drawn from the *Pathways in ASD* study, an inception cohort of 421 families recruited across five provinces in Canada, aimed at better understanding developmental trajectories in autism. The study was approved by each of the local Research Ethics Boards and informed consent was obtained from all participating families. Children in the cohort met ASD criteria on the Autism Diagnostic Observation Schedule (ADOS; [33]), on the Social and at least one other domain on the ADI-R [27], as well as DSM-IV criteria (APA, [34]) according to expert clinical judgement. Exclusion criteria included any known genetic abnormality, neuromotor disorder (such as cerebral palsy), or severe hearing or vision disorders. Only one child per family with ASD was included.

The ADI-R was administered at three time points (time of diagnosis = T1; age 6 years = T2; age 11 years = T3). In the current study, we included all participants with at least the first ADI-R time point and a valid NVIQ, administered at age 8 (n = 205). The Perceptual Reasoning Index (PRI) of the Wechsler Intelligence Scales for Children (WISC-IV; Wechsler, 2003) was used as a proxy of NVIQ (see WISC-IV section below and Table 1 for more details and Additional file 1: Table S1 for site distribution).

**Table 1:** Number of participants, age and scores for each measure and time point

Measure	Age of Dx (T1)	Age 6 (T2)	Age 8	Age 11 (T3)
N	205	199	205	157
Age	3.43 (.75)	6.61 (.33)	8.73 (.21)	10.73 (.21)
ADI-R RRB	4.52 (2.30)	4.07 (2.57)	–	3.66 (2.35)
WISC-IV PRI	–	–	91.28 (19.50)	–

*Note.* Numbers in parenthesis are standard deviations. Dx = Diagnosis. ADI-R = Autism Diagnostic Interview -Revised. Numbers reported are mean total scores for the RRB domain using current scores. WISC-IV PRI = Wechsler Intelligence Scales for Children- Fourth Edition, Perceptual Reasoning Index. Number reported is the mean Standard Score

## Measures

### Autism diagnostic interview-revised (ADI-R)

The ADI-R [27, 35] is a comprehensive caregiver interview, administered by a trained clinician. It is one of the gold standard instruments used to discriminate between children with and without autism [36] and to differentiate between ASD and an intellectual disability or a language impairment [27]. It covers the different domains of autistic symptoms, one of which is the Restricted and Repetitive Behaviors domain. In total, 15 ADI-R items relevant for RRB were included in the current analysis (see Additional file 1: Table S2 for a list). During the ADI-R interview, parents indicated whether the behavior was present in the past, «ever», and if it was present at time of administration, «current». Given the longitudinal design of the study, only the «current» scores of the RRB items were included in the analysis. Measurement invariance over time has been previously demonstrated for the ADI-R [37].

Scores on the ADI-R items vary between 0 and 3, where 0 indicates the absence of the behavior or no abnormality, 1 indicates the behavior is present or abnormal to some degree, 2 indicates the behavior is present and frequent/severe/abnormal enough to impair functioning to some degree and 3 indicates the behavior is present and is sufficiently intrusive or abnormal to significantly impair functioning. In line with what was done by Bishop, Richler [2] to examine prevalence separately from severity, a presence/absence score was computed by recoding scores of 2 and 3 into «1». All included ADI-R items were therefore transformed into binary variables where «0» indicates the behavior is absent and «1» indicates it is present. As the current study is a first attempt to study individual RRB using a longitudinal design and because the examination of severity in relation to NVIQ and age in Bishop’s 2006 study did not yield any additional information, the analysis considers only the presence/absence of a behavior.

The ADI-R scoring system typically recodes scores of ‘8’ as ‘0’, indicating that no atypicality was observed. However, this conflates children for whom RRB could have been observed but were not, with those who did not yet have the linguistic ability to show certain RRB. Coding these children’s scores of ‘8’ as missing is therefore the more conservative approach in that it does not assign the presence or absence of RRB to these children. Therefore, items with scores of ‘8’ were recoded in the following manner. For *Stereotyped Speech* and *Verbal Rituals* children who were coded ‘8’ (i.e., they did not have enough phrase speech to make assessment possible) were treated as missing in this analysis. Similarly, for *Circumscribed Interests*, children below the age of three who were coded as ‘8’ were recoded as missing. In addition, for *Unusual Attachment to Objects*, children who were coded as ‘6’ or ‘7’ (i.e., “attachment to usual objects after 5 years old or to such intensity that it interferes with social functioning” or “series of short lasting attachment to unusual objects”) were recoded into ‘1’ as these indicate a certain level of atypicality in the behavior, despite not being considered in the ADI-R algorithm [35].

### Wechsler intelligence scale for children—fourth edition (WISC-IV)

The standard score obtained on the Perceptual Reasoning Index (PRI) of the WISC-IV was used as a proxy for NVIQ. This test was administered at 8 years and was determined to be the best available proxy for NVIQ for this cohort. Tests administered at previous time points are more prone to be influenced by testability issues (i.e., difficulties following directions, understanding spoken language, etc.) and therefore might not be as representative of non-verbal reasoning abilities [38]. Including the latest time-point for which IQ was available was also motivated by the fact that IQ is more stable in later childhood (Flanagan et al., 2015).

### Statistical analysis

Data were analysed with binary logistic Generalised Estimating Equations (GEE) in Stata v15 [39]. GEE was chosen as it: 1) accounts for missingness (i.e., GEE uses all available data from each child, and accounts for selective attrition associated with included covariates and factors); 2) is robust to the misspecification of the pattern of correlation among ADI-R items through the use of robust standard errors for the calculation of the Wald statistics; and 3) allows flexible inclusion of age as a time-varying covariate, which represents both age at enrollment between participants, as well as within- participant change in RRB over time. Models had a logit link function and an exchangeable correlation matrix with robust standard errors; binary ADI-R score was the outcome. The first model included main effects of ADI-R item as a multivariate measure [33, 39], IQ at T3, Age (of the child at each time point, included as a time varying covariate) and controlled for Site (Montreal, Halifax, Hamilton, Vancouver, Edmonton) and age at enrollment. The following two- and three-way interactions were added as a secondary step: ADI-R item*Age, ADI-R item*NVIQ, ADI-R item*Age*NVIQ. To break down the main effect of ADI-R item, separate GEE models were then run for each ADI-R item, including main effects of NVIQ, Age and Site; an Age*NVIQ interaction was added as a second step. The testparm command in Stata was used to assess the overall significance of main effects in the model. For the analysis split by ADI-R item, two types of results are reported. First, given that we had no prior hypothesis regarding any specific ADI-R item’s link with age and/or NVIQ, Bonferroni corrections were deemed appropriate (adjusted level of significance, 0.05/15 = 0.0033) However, this is a highly conservative approach that could lead to false negatives, thus we also reported Benjamini–Hochberg corrected results using a False Discovery Rate (FDR) of 0.05. For a complete list including non-corrected results for all items, see Additional file 1: Table S2. As a sensitivity analysis, the same analyses were re-run using verbal IQ instead of NVIQ (see Additional file 1: Table S3).

## Results

### Effect of age

**Fig. 1:**
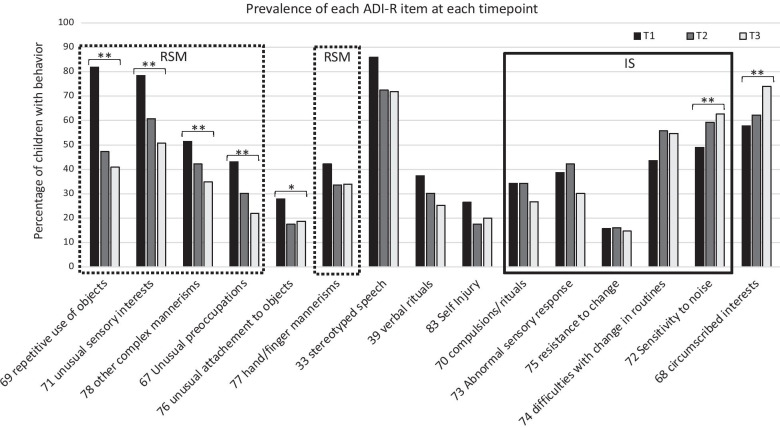
Prevalence of each ADI-R item at each time point. ** = item with a significant effect of age after Bonferroni corrections. * item with a significant effect of age after Benjamini–Hochberg corrections. T1 = time of Diagnosis, T2 = 6 years old, T3 = 11 years old. RSM dotted Boxes = items from the Repetitive Sensory Motor factor, IS Black box = items from the Insistence on sameness factor

Results from the main GEE model showed a significant main effect of age (Wald χ^2^(1) = 28.40, *p* < 0.001) such that the overall presence of ADI-R RRB decreased over time across the whole sample. There was also a significant interaction between age and ADI-R item (Wald χ^2^ (14) = 160.74, p < 0.001, See Table 2). Separate GEE models for each ADI-R item, using a Bonferroni correction for multiple comparisons, showed that this interaction was driven by certain RRB significantly decreasing with age (*Unusual Sensory Interest*, *Repetitive Use of Objects, Other Complex Mannerisms* and *Unusual Preoccupations*) whereas others significantly increased with age (*Circumscribed Interests*, *Sensitivity to Noise).* Benjamini–Hochberg corrections led to the identification of one additional item that significantly decreased with age (*Unusual Attachment to Objects*) (see Fig. 1). The dotted box in Fig. 1 identifies items that consistently loaded onto the RSM factor in factor analyses of the ADI-R RRB items, and the black box represents those that loaded onto the IS factor [4, 40, 41]. As for the unboxed items, they either consistently did not load on any factor (*self-injury*), inconsistently loaded onto either RSM or IS or no factor (*Unusual attachment to objects*) or load onto a third factor (*Stereotyped speech* and *Verbal rituals* as well as *Circumscribed interests*) [4, 40, 41]. For a visual representation of how the presence/absence scores were separated into scores of 1–2 and 3, see also Additional file 1: Figure S1, which presents the percentage of children for whom scores of 1–2 or 3 were endorsed at each timepoint for each item.

**Table 2:** GEE model results

ADI-R item	Wald χ^2^ (14) = 624.32, *p* < 0.001
Site	Wald χ^2^(4) = 25.87, *p* < 0.001
Age at enrollment	Wald χ^2^( 1) = < 0.01, *p* = 0.989
Age	Wald χ^2^(1) = 28.40, *p* < 0.001
NVIQ	Wald χ^2^(1) = 2.70, *p* = 0.101
ADI-R item * Age	Wald χ^2^ (14) = 160.74, p < 0.001
ADI-R item * NVIQ	Wald χ^2^ (14) = 30.22, p = 0.007
ADI-R item * Age * NVIQ	Wald χ^2^(15) = 32.60, *p* = 0.005

### Effect of NVIQ

**Fig. 2:**
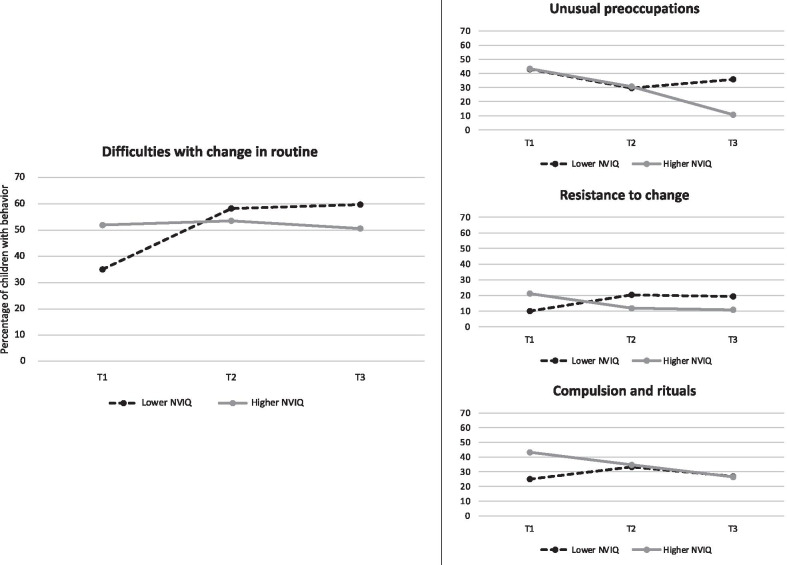
Percentage of children at each time point for each item presenting a significant interaction between age and NVIQ. The item on the left was significant following Bonferroni correction and the three additional items on the right were significant only when using Benjamini–Hochberg corrections. Lower NVIQ =  < 93; *n* = 101, Higher NVIQ =  > 93; *n* = 104. T1 = time of Diagnosis, T2 = 6 years old, T3 = 11 years old

There was no main effect of NVIQ (Wald χ^2^(1) = 2.70, *p* = 0.101) in the main GEE model. However, there was a significant 2-way interaction between NVIQ and ADI-R item (Wald χ^2^ (14) = 30.22, p = 0.007). There was also a 3-way interaction between NVIQ, age, and ADI-R items (Wald χ^2^ (15) = 32.60, *p* = 0.005). See Table 2 for the detail of all the model results and results for covariates. Separate GEE models, split by ADI-R item and level of significance corrected for multiple comparisons using Bonferroni, showed a significant interaction between age and NVIQ only for *Difficulties with Change in Routine*, which increased over time in children with lower NVIQ. Benjamini–Hochberg corrected results include three additional items showing a significant interaction between age and NVIQ (*Unusual Preoccupations*, *Resistance to Change* and *Compulsions and Rituals*). All of these items except *Unusual preoccupations*, which usually loads onto the RSM factor, typically loaded onto the IS factor when using factor analysis on the ADI-R [4, 40, 41]. Even though NVIQ was included in the model as a continuous variable, to allow for a graphic representation, IQ groups were split according to the median (Median standard score of 93, Range 51–134) and are presented in Fig. 2. We also split the sample into three groups based on clinical cut-off (one SD below the mean (< 85), versus average NVIQ (85–115) versus one SD higher than the mean (< 114), and included these graphs in supplementary material for reference (see Additional file 1: Figure S2).

Analysis with verbal IQ led to a similar pattern of results with significant 2- and 3-way interactions and no significant main effects for VIQ (see Additional file 1: Table S3). However, when broken down by ADI-R item and corrected for multiple comparisons, there were no significant main effects for VIQ and there was a significant age*VIQ interaction only for item 39 (*Verbal rituals*), such that the prevalence of this item tended to diminish with age in children with higher VIQ, but remained stable for those with lower VIQ during the same time period (see Additional file 1: Figure S3). This suggests that the item-level effects may be somewhat specific to NVIQ.

## Discussion

We conducted a novel longitudinal investigation of individual Restricted and Repetitive Behaviors in a cohort of autistic children followed from preschool age of diagnosis to 11 years of age. The association between the presence or absence of each of the 15 RRB items from the ADI-R and age as well as NVIQ was explored. First, we found a main effect of age, driven by decreasing prevalence for four RRB throughout childhood (*Unusual Sensory Interest*, *Repetitive Use of Objects, Other Complex Mannerisms* and *Unusual Preoccupations*). Other items did not significantly change with age and only two (*Sensitivity to Noise* and *Circumscribed Interest*) for which prevalence increased significantly. We found no main effect of NVIQ on the prevalence of any of the ADI-R items. There was however a significant interaction between *Difficulties with Change in Routine*, age and NVIQ, indicating that the change in the prevalence of these behaviors with age depended on children’s NVIQs. Less conservative corrections led to three additional items showing a significant interaction with age and NVIQ, namely *Unusual Preoccupations*, *Resistance to Change* and *Compulsion and rituals.* These results illustrate that when examining RRB, item-level analyses may be helpful before grouping behaviors into higher level domains, as their associations with age and intelligence are complex and heterogeneous. and the associations do not perfectly map onto factors derived from factor analysis. Furthermore, results appeared to be somewhat specific to NVIQ and were not replicated using VIQ.

The association that we found between age and the prevalence of RRB parallels the decrease observed from preschool age to school age when considering all behaviors included in the RRB domain as a single construct [22, 42, 43] or when studying the change with age on the RSM factor derived from the ADI-R [4, 28]. However, the item-by-item analysis used in the current study demonstrates that this decrease is not present across all RRB nor do the items which show change consistently load onto the RSM factor. When using Bonferroni corrections, four items showed a decrease over time, all of which typically load onto the RSM factor. However, the *Hand and finger mannerisms* item, which also typically loads onto the RSM factor, did not show change over time. Two items showed an *increase* over time. One of these, *Sensitivity to noise*, typically loads onto the IS factor, but no other items associated with the IS factor showed change over time (see Fig. 1). The other item that increased over time was *Circumscribed interest*, which often constitutes its own factor. The use of less conservative correction led to only one additional item significantly decreasing with age (*Unusual attachment to objects)*, an item that loads sometimes onto the RSM factor, onto the IS factor or not on any factor [4, 40, 41]*.* Given that our measure of prevalence was based on absence or presence of the behavior, the significant effect of age indicates that for those items, a significant proportion of children went from presenting the behavior (scores of 1–2 or 3) to not presenting it at all (score of 0) or the opposite. Such changes are not only statistically significant, but also of high clinical importance as they entail shifts from presenting an atypical behavior to presenting no detectable atypicality or from a typical behavior to an atypical one for a significant portion of the group. Taken together, the current results suggest that, to understand the developmental pathways of RRB in autism, an item-based approach is warranted, at least as a first step.

The differential effect of age on the prevalence of RRB during childhood could be the result of some behaviors morphing into other behaviors. For example, as the child gets older, *Repetitive Use of Objects* could transform to become a *Circumscribed Interest* [44]. This hypothesis is consistent with our results as well as with Bishop and colleagues’ previous findings that *Repetitive use of object*s tends to diminish with age while *Circumscribed Interests* tends to increase [2]. To directly test this hypothesis that RRB change throughout development, studies using latent transition models are needed. It is also possible that some RRB represent a step of the autistic development and are therefore transient. For example, in a similar hypothesis regarding language development in autism, it was proposed that *Verbal Rituals* and *Stereotyped Speech* such as echolalia are milestones of language development and thus are more frequent when the child first starts speaking, and diminish and disappear as the child develops more oral language [13, 45, 46]. As for the increased prevalence of *Sensitivity to Noise* during childhood it is possible that, as children get older, they are exposed to a greater diversity of environments that might trigger their sensitivity, such as school classrooms, corridors and cafeterias, which are typically noisier and less controlled than those in daycare or home settings. It is plausible that parents of children who are sensitive to noise, consciously or not, tend to adapt the environment to avoid some triggers and, as such, a lot of the noise the child is exposed to is self-generated, which usually does not trigger sensitivities or adverse reactions [47]. It is also interesting to note that this item generally loads onto the IS and not the RSM factor despite its sensory component. It is the only item from this IS factor for which prevalence significantly increased with age, warranting granular investigation of this specific behavior.

With respect to NVIQ, while we found no significant main effect on the prevalence of the ADI-R items, NVIQ did interact with age for one specific behavior, *Difficulties with Change in Routine*. This result is consistent with results from Bishop’s cross-sectional study [2], which found differences between NVIQ groups in the prevalence of certain RRB only in older children, except for *Compulsion and Rituals* that differed between NVIQ groups only at earlier ages (3–6 years). In the present study, *Difficulties with Change in Routine* followed a path characterized by the behavior being less prevalent in children with lower NVIQ at age of diagnosis and becoming more prevalent as they grew older, whereas in children with higher initial NVIQ, it remained stable during the same time. The path for three additional items (*Resistance to Change* and *Compulsion and rituals* and *Unusual preoccupation*s) was similar and significant when using Benjamini–Hochberg corrections, but was characterized by a decrease in prevalence with age in children with higher NVIQ (instead of stability), whereas the prevalence was stable or slightly increased in children with lower NVIQ. All of the behaviors that significantly interacted with NVIQ and age consistently loaded onto the IS factor, except *Unusual preoccupation*s which loads more often on the RSM factor. This could indicate that, as has been suggested by other authors [4], some IS behaviors and potentially some RSM behaviors may be more directly associated with prototypical autistic development, whereas other behaviors may distinctly map onto developmental trajectories associated with other conditions or comorbidities such as ID.

In typical development, it has been suggested that rituals, compulsive-like behaviors and sameness behaviors help children adapt to and to gain a sense of control over their environment [48]. It is therefore possible that such behaviours play a similar role in autistic development. Hence, these behaviors may represent a key step in autistic development, with a peak in prevalence at a certain age followed by diminution in children without ID, whereas this pattern might happen later or not at all in autistic children with lower NVIQ [2].

These results highlight the importance of considering age when investigating the association between RRB and cognitive level. RRB can be observed in autistic children of all IQ levels, but they tend to vary in degree and may change or disappear with age. Our results also show that investigation of individual behaviors over time is important to understand which behaviors are sensitive to developmental change and how this change relates to factors such as cognitive abilities. Clinically, our results could also have implications for intervention recommendations. For example, intervention will not be needed if a specific behavior is a feature of autistic development and will naturally diminish with age in children with typical NVIQ. The same logic could be applied to behaviors that tend to increase with age in autistic children with typical or high NVIQ, as these may have a positive impact on development and learning, like they have in typically developing children [14, 15, 19]. For example, some RRB, like circumscribed interests, could be encouraged as a strategy to promote learning in autism [44, 49, 50]. Finally, other behaviors which are more prevalent in autism than in other conditions, but do not seem to be a feature of autistic development per se, such as self-injury [51], warrant individualized investigation and intervention to limit their occurrence.

## Limitations

Our findings need to be considered against several inherent limitations related to measurement of RRB in autism. Indeed, here we focused solely on the presence or absence of RRB as measured through the ADI-R, which led to an investigation of the change in prevalence of each RRB, without accounting for either frequency or severity of each behavior. Using the severity scores from the ADI-R, which represent a measure of perceived functional impact and not necessarily of frequency, Bishop, Richler [2] had shown that results were either similar to when using presence/absence scores or were not significant. They concluded that age and NVIQ were more indicative of whether a child would present a behavior, than of the functional impact of this behavior. Kim and Lord [13], using the Autism Diagnosis Observation Schedule (ADOS-G; 33) found severity of RRB to be independent of age. As a future step, it would be interesting to investigate whether changes in severity, measured in terms of frequency of behavior, parallel those we found in prevalence. However, the field of autism is lacking a good measure of RRB frequency that is independent of functional impact. Only observational studies provide such measures of frequency/duration [23, 52]. The use of multiple measures and informants as well as the development of new measures is therefore warranted to strengthen this area of research. The results of this study also need to be interpreted while taking into consideration that the ADI-R is a parent-reported measure. It is therefore possible that some behaviors were present, but not noted or noticed by parents.

Other limitations include the measurement of intelligence in autism. Intelligence measured through conventional IQ tests tends to underestimate the intellectual potential of most autistic children [38, 53]. The choice to use PRI at age 8 as a measure of NVIQ in this study aimed at minimizing the effect of testability and reducing the risks of underestimation. It is however still possible that the NVIQ of certain of our participants remained underestimated, especially in the children who were less verbal and for whom intelligence testing is often more challenging [54, 55].

Finally, another limitation is that we did not investigate the association between age and RRB in relation to other developmental indicators, such as adaptive abilities, nor did we investigate the relation with sex, given the very small number of girls in our sample (n = 28). Our knowledge of RRB in autistic girls is sparse, but a recent study investigating RRB in relation to age and NVIQ found evidence suggesting a differential effect of NVIQ on RRB in autistic boys and girls. As the prevalence of RRB and their link with age and cognitive abilities might differ in autistic girls and boys, investigating sex would be an interesting future step. In addition, future studies following the development of RRB in children after 11 years old and into adolescence and adulthood could further inform those associations. Investigating stability and change in later development would allow us to investigate the transient or permanent nature of specific RRB in autism development. For example, it would help determine whether the presence of certain RRB in children with lower NVIQ follows the same pathway as in children with higher NVIQ but with a delay, or whether it persists into adolescence and adulthood in these children.

## Conclusions

This study confirms previous findings showing that when taken as a whole RRB tend to diminish or even disappear from age of diagnosis to later childhood, especially in autistic children with higher NVIQ. However, not all RRB follow this trajectory. Indeed, although some RRB diminish over time, others increase in prevalence with age, and these changes vary as a function of NVIQ. Some RRB may be an inherent part of autistic development, but more research is needed to fully understand how each evolves over time, and how this evolution is associated with other aspects of development.

## Supplementary Information

**Additional file 1**. Supplementary file 1.

## Data Availability

Participants did not consent to making their data publicly available and it would be a violation of our ethics to openly share this confidential dataset. However, if researchers are interested in collaborating or learning more about Pathways in ASD data they can contact the corresponding author. The dataset analysed during the current study is not publicly available due to the data analysed being composed of sensitive private information that could compromise the privacy of the research participants, but is available from the corresponding author on reasonable request.
