# Supplementary material for: Non-verbal IQ and change in restricted and repetitive behavior throughout childhood in autism: a longitudinal study using the Autism Diagnostic Interview-Revised
Source: Mol Autism. 2021 Aug 14;12:57. doi: 10.1186/s13229-021-00461-7 (PMC8364071; doi:10.1186/s13229-021-00461-7)
Supplement: Supplementary file 1 — Additional file 1. Supplementary file 1. [file 13229_2021_461_MOESM1_ESM.docx]

Table S1. Site distribution

| Site | N | Percentage |
| --- | --- | --- |
| Halifax | 28 | 13.7 |
| Montreal | 76 | 37.1 |
| Hamilton | 24 | 11.7 |
| Vancouver | 54 | 26.3 |
| Edmonton | 23 | 11.2 |
| Total | 205 | 100 |

Table S2. *P* values for main effects of Age and NVIQ and interaction between Age and NVIQ for each ADI-R item.

| # | Description | Age | | NVIQ | | Age*NVIQ | |
| --- | --- | --- | --- | --- | --- | --- | --- |
|  |  | Wald χ^2^ (1) | *p* | Wald χ^2^ (1) | *p* | Wald χ^2^ (1) | *p* |
| 83 | Self-Injury | 2.98 | 0.0845 | 4.56 | 0.0327* | 0.09 | 0.7680 |
| 72 | Sensitivity to noise | 9.01 | 0.0027*** | 0.74 | 0.3904 | 2.43 | 0.1193 |
| 73 | Abnormal response to sensory stimuli | 3.57 | 0.0589 | 1.12 | 0.2907 | 0.93 | 0.3350 |
| 71 | Unusual Sensory Interests | 35.66 | 0.0000*** | 6.48 | 0.0109* | 1.70 | 0.1925 |
| 69 | Repetitive use of Object | 71.99 | 0.0000*** | 5.71 | 0.0169* | 2.01 | 0.1562 |
| 77 | Hand and finger mannerisms | 1.47 | 0.2259 | 7.84 | 0.0051* | 0.08 | 0.7736 |
| 78 | Other complex mannerisms | 11.28 | 0.0008*** | 4.86 | 0.0275* | 0.46 | 0.4971 |
| 33 | Stereotyped speech | 4.50 | 0.0339** | 2.59 | 0.1075 | 1.38 | 0.2394 |
| 39 | Verbal Rituals | 3.29 | 0.0698 | 1.07 | 0.3007 | 3.95 | 0.0469* |
| 74 | Difficulties with change in routines | 4.43 | 0.0353* | 0.15 | 0.7025 | 9.35 | 0.0022*** |
| 75 | Resistance to change | 0.20 | 0.6550 | 0.03 | 0.8564 | 7.95 | 0.0048** |
| 70 | Compulsions/rituals | 2.86 | 0.0908 | 0.39 | 0.5346 | 7.13 | 0.0076** |
| 67 | Unusual preoccupations | 16.99 | 0.0000*** | 4.86 | 0.0275* | 8.44 | 0.0037** |
| 76 | Unusual attachment to objects | 7.01 | 0.0081** | 1.65 | 0.1986 | 0.90 | 0.3435 |
| 68 | Circumscribed interests | 11.61 | 0.0007*** | 0.75 | 0.3862 | 0.37 | 0.5428 |

Note. *significant when uncorrected (<0.05). **Significant following Benjamini-Hochberg correction with a False Discovery Rate of 0.05. *** significant following Bonferroni corrections (<0.00333).

Table S3. GEE Model results for Verbal IQ (VIQ)

| ADI-R item | Wald χ^2^ (14) = 669.21 *p* < 0.001 |
| --- | --- |
| Site | Wald χ^2^(4) = 24.32, *p* < 0.001 |
| Age at enrollment | Wald χ^2^(1) = 0.00, *p* < 0.947 |
| Age | Wald χ^2^(1) = 28.81, *p* < 0.001 |
| VIQ | Wald χ^2^(1) = 0.32, *p* = 0.574 |
| ADI-R item * Age | Wald χ^2^ (14) =164.72, p <0.001 |
| ADI-R item * VIQ | Wald χ^2^ (14) = 40.16, p <0.001 |
| ADI-R item * Age * VIQ | Wald χ^2^(15) = 111.82, p <0.001 |

Note. Decomposition of the ADI-R item by Age interaction leads to no significant main effect of age on any ADI-R items following Bonferroni corrections. Decomposition of the three-way interaction leads to item 39 (*Verbal rituals*) significantly interacting with age and VIQ.

Figure S1.

Figure S1. Percentage of children endorsing scores of 1-2 or 3 at each timepoint for each item.

Figure S2.

Figure S2. Percentage of children at each time point for each item presenting a significant interaction between age and NVIQ. The item on the left was significant following Bonferroni correction and the three additional items on the right were significant only when using Benjamini-Hochberg corrections. Groups were separated into below mean NVIQ = <85; *n*=79, Normal NVIQ = 85 to 114; *n*=99, Higher NVIQ= >114; *n*= 27. T1= time of Diagnosis, T2= 6 years old, T3= 11 years old.

Figure S3. Percentage of children showing verbal rituals (item 39) at each time point for low VIQ (lower than 85, n=83) and high VIQ (85 and higher n=122).
